# Increasing the Performance of {[(1-x-y) LiCo_0.3_Cu_0.7_] (Al and Mg doped)] O_2_}, xLi_2_MnO_3_, yLiCoO_2_ Composites as Cathode Material in Lithium-Ion Battery: Synthesis and Characterization

**DOI:** 10.3390/mi14020241

**Published:** 2023-01-18

**Authors:** Sara Shahriari, Fatemeh Mollaamin, Majid Monajjemi

**Affiliations:** 1 Department of Chemistry, Central Tehran Branch, Islamic Azad University, Tehran P.O. Box 1496969191, Iran; 2 Department of Biomedical Engineering, Faculty of Engineering and Architecture, Kastamonu University, 37100 Kastamonu, Turkey; 3 Department of Chemical Engineering, Central Tehran Branch, Islamic Azad University, Tehran P.O. Box 1496969191, Iran

**Keywords:** lithium-ion batteries, Al doping, Mg doping, cathode materials, LiCoO_2_

## Abstract

Twenty-eight samples of {[(1-x-y) LiCo_0.3_Cu_0.7_](Al and Mg doped)]O_2_}, xLi_2_MnO_3_, and yLiCoO_2_ composites were synthesized using the sol–gel method. Stoichiometric weights of LiNO_3_, Mn(Ac)_2_⋅4H_2_O, Co(Ac)_2_⋅4H_2_O, Al(NO_3_)_3_.H_2_o, Mg(NO_3_)_2_⋅6H_2_O, and Cu(NO_3_)_2_.H_2_O for the preparation of these samples were applied. From this work, we confirmed the high performance of two samples, namely, Sample 18, including Al doped with structure “Li_1.5_Cu_0.117_Co_0.366_Al_0.017_Mn_0.5_O_2_” and Sample 17, including Mg doped with structure “Li_1.667_Cu_0.1_Mg_0.017_Co_0.217_Mn_0.667_O_2_”, compared with other compositions. Evidently, the used weight of cobalt in these two samples were lower compared with LiCoO_2_, resulting in advantages in the viewpoint of cost and toxicity problems. Charge and discharge characteristics of the mentioned cathode materials were investigated by performing cycle tests in the range of 2.2–4.5 V. These types of systems can help to reduce the disadvantages of cobalt arising from its high cost and toxic properties. Our results confirmed that the performance of such systems is similar to that of pure LiCoO_2_ cathode material, or greater in some cases. The biggest disadvantages of LiCoO_2_ are its cost and toxic properties, typically making it cost around five times more to manufacture than when using copper.

## 1. Introduction 

Transition metals in the second row of Mendeleev’s table, such as manganese, copper and cobalt, are suitable as cathode materials in lithium-ion batteries (LIBs), due to their potential for operating in high voltages and large energy storage in small spaces. Although the cobalt in cathode materials, such as LiCoO_2_ salt, gives rise to high capacity for storing energy, it is expensive and toxic, with a propensity toward destructive reactions and explosion with the electrolyte. In Table 1, both advantageous and disadvantageous factors for several cathode materials in lithium-ion batteries (LIBs) are listed [1,2,3,4,5].

This is a major problem when considering manufacturing in huge commercial items, in which decreasing the extra costs is an important issue. Safety consideration is also a subject of crucial importance to LIBs, as this problem appears more so in big size batteries compared with small size batteries. For example, batteries for electrical vehicles would have a higher risk of catching fire compared with batteries for cell phones. The major advantage of using a solid solution instead of liquids in cathode materials is that the explosion of batteries can be controlled. This is especially true when using a mixture of doped metals, such as Mg and Al, in the structure of composites. Moreover, {[(1-x-y) LiCo_0.3_Cu_0.7_] (Al and Mg doped)] O_2_} and xLi_2_MnO_3_, yLiCoO_2_ composites, containing Al and Mg doped inside the cathode materials structures, are more environmentally friendly when compared with isolated cathodes such as LiCoO_2_ [4,5].

**Scheme 1:**
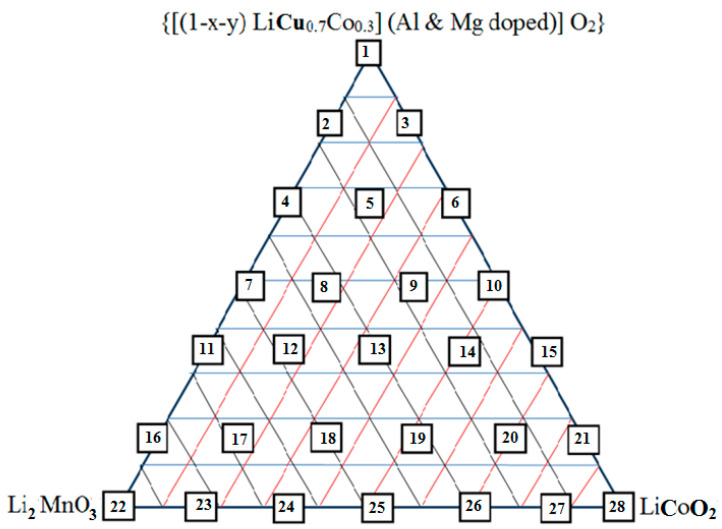
Pure, binary, and ternary diagram of Li_2_MnO_3_, LiCoO_2_, and {[(1-x-y) LiCu_0.7_Co_0.3_] (Al and Mg doped)] O_2_}.

The aim of this investigation is to prepare a ternary solid solution of Li-based cathode material, containing a solid mixture of cobalt, manganese, copper, aluminum, and magnesium, which are advantageous replacements for LiCoO_2_, which is expensive and toxic [6]. Therefore, a ternary composition diagram containing (1-n-m) LiCu_0.7_Co_0.3_O_2_, nLi_2_MnO_3_, and mLiCoO_2_ was drawn for calculating 28 samples with various mole fractions of these three compounds. Similar to the work of Rossen [6], an investigation was conducted by Saphr et al. [7] in the viewpoint of electrochemical function, which indicated that Ni and Cu as active ions and Mn were present in “+2” and “+4” oxidation instead of “+3” and “+3”, respectively. They confirmed that at a temperature of 700 K, there were large failing capacities after 30 cycles from 155 mAh/g to 120 mAh/g, and after 35 cycles, the failing capacity was around 70 mAh/g. Copper, which forms between +1 and +2 valence positions, is an active material in this cathode composition, while manganese is +4 and remains without Jahn–Teller distortion with the +3 valance (Mn^+3^). Makimura and coworkers [8] presented an initial discharging around 151.6 mAh/g for 30 cycles at 4.4 V, and, again in 2003 [9], they exhibited 200 mAh/g of rechargeable capacities for Co/Cu/Mn mixtures among the voltages between 2.5 and 4.5 V after 35 cycles [9]. It is clear that the precise mixing of weighted percentages of various transition elements gives advantageous efficiency. Therefore, these materials are derived from Li (Ni (1-x-y) Mn_x_ Co_y_) O_2_ categories that were first published in 1999 and 2000 by Liu and Yoshio, respectively [10,11]. It can be demonstrated that extra cobalt would stabilize the ternary solid mixtures and block the Cu from entering inside the lithium layers, and in contrast, Cu and Mn would provide the structural stabilities and high capacities together, respectively. Since an excess of cobalt causes a decrease in capacities, while large amounts of copper and manganese cause problems with mixing and in spinel structures, the mixing of these elements must therefore be optimized with a suitable mole fraction. In addition, doping some main group metals, such as Mg and Al, will remove these problems due to their high electro-positivity, automatically. In 2001, Makimura and coworkers synthesized the layered compound of LiNi_1/3_Mn_1/3_Co_1/3_O_2_ [12] and showed that this cathode has about 200 mAh/g reversible discharge capacity in the range of 2.65–4.55 V, including large rate capabilities and thermal stabilities. For accurate stoichiometry in the mixture of Li (Cu_x_Co_(1 -2x)_ Mn_x_)O_2_ and x = 1/3 [13,14,15], the expected oxidation numbers must be +3 (such as Mn^+4^, Co^+3^, and Cu^+2^), with electrochemical flow within the range between 2.5 and 4.4 volts versus lithium ions (through two electron transfer reactions between Co^+3^/Co^+4^ and Cu+/Cu^2^+). Studies of LiCu_0.4_Mn_0.4_Co_0.2_O_2_, Li (Cu_0.8_Co_0.2_)O_2_, and LiCu_0.8_Co_0.15_Al_0.05_O_2_ cathodes have been conducted. Although the rate capabilities of these types of material are smaller than that of LiCoO_2_, their thermal stabilities are much better. In this study, we concentrated on various compositions of pure, binary, and ternary solid solutions containing Li_2_MnO_3_, LiCoO_2_, and {[(1-x-y) LiCu_0.7_Co_0.3_] (Al and Mg doped)] O_2_} (Scheme 1). Currently, Li_2_MnO_3_ has been determined for its excellent stability, high degree of safety, non-toxicity, and cheaper cost compared with LiCoO_2_ [16]. Li_2_MnO_3_, which can be considered as Li [Li_1/3_Mn_2/3_] O_2_, has a similar structure to that of LiCoO_2_ with a small difference in layers due to the super-lattice ordering of Li^+^ and Mn^4+^ in transition metal layers.

Li_2_MnO_3_ has a rock-layer shape including lithium and manganese ions in alternating (1:2 ratio, respectively) positions, and the layers are divided via several cubic oxygen layers. Since manganese is present in +4 oxidation, it is impossible to be further oxidized in low voltages and, therefore, remains as Li_2_MnO_3_, whereas in higher voltages, i.e., >4.3 volt, this material will extract the Li_2_O from behind the MnO_2_ layers. It is notable that the mentioned mechanism is one-way and completely irreversible, and, consequently, the Li+ ion can return during discharging toward LiMnO_2_, which remains as an active oxide. Lithium atoms that are broken from Li_2_O during charging can be attached to the higher position of metals capacities. The use of cathodes with Li_2_MnO_3_ is important in simplifying the intercalation mechanism, as well as providing structural stabilities during this intercalation [17]. Solid solutions are fundamentally determined in the viewpoint of obtaining a suitable cathode composition with the highest functionality to increase the performance of cyclability, capacity, safety, voltages, amperages, and structural stabilities while also having low costs and no problems with toxicity [18,19]. Li_2_MnO_3_ cathodes, as suitable solid solutions, were found for two main reasons: the extraction of two Li+ ions at voltages >4.4 V, which gives the extra amount of initial charge capacities; the MnO_2_ component, which when omitted of Li, gives strong structural stability in this composition. Not only does the initial crystal of the cathode material have specific capacity, but it also adheres to the condition of structural stabilities, which is suitable for any increase in the cycle life. During lithium-ion extraction, copper and cobalt will be oxidized from +1 to +3 valance positions toward +2 to +4, respectively; therefore, the crystal cell volume will be changed. Al as a substitution in cobalt structures is a suitable doped metal for improving stability and electrochemical performance. It is notable that aluminum remains in +3 oxidation situations, which has no effect on its capacity and only helps to reduce the structure volume of composites, which allows for the structure of cathode materials during any number of cycles to be maintained. Consequently, in the composition of (1-x-y) LiCu_0.7_Co_0.2_Al_0.1_, xLi_2_MnO_3_, and yLiCoO_2_ (Table 2), the high level of copper generally gives higher initial specific capacities, while cobalt and aluminum improve the structural stabilities and life cycles. Not only does Mg-doping improve the capacity [20], but it also causes the insertion of Li+ in the (1-x-y) LiCu_0.6_Co_0.3_Mg_0.1_, xLi_2_MnO_3_, and yLiCoO_2_ composition. Finally, Mg doping removes the phase transitioning problem that usually occurs in LiNiO_2_ during cycling, and this is an advantage of Mg doping for the charge/discharge reversibility in any type of these compositions.

## 2. Materials and Methods

### 2.1. Cathode Electrode Preparation

The composites were synthesized using the sol–gel method due to simple chemical reactions at a low temperature. Stoichiometric weights of the LiNO_3_, Li_2_MnO_3_, Mn(Ac)_2_⋅4H_2_O, Co(Ac)_2_⋅4H_2_O, Al(NO_3_)_3_⋅H_2_O Mg(NO_3_)_2_⋅6H_2_O, and Cu(NO_3_)_2_⋅H_2_O as derived materials of lithium, iron, manganese, cobalt and copper, in 28 samples of {[(1-x-y) LiCo_0.3_Cu_0.7_](Al and Mg doped)]O_2_}, xLi_2_MnO_3_ and yLiCoO_2_ composites, were used (Table 2 and Table 3). These mixtures were first dissolved in 55 mL of DI H_2_O, and then equivalent-molar weights of citric acid were added. The electrolytes were prepared by dissolving one mole LiPF_6_ in a blend of ethylene carbonate and dimethyl carbonate. Ethyl methyl carbonate in a dry glove box was filled with high pure argon. The electrodes were prepared by mixing 75 wt% Li Cu(NO_3_)_2_/C powder, 15 wt% acetylene black, and 10 wt% polyvinylidene fluoride, and this process was continued by coating the slurry onto Al foil current collector and dyeing at 130 °C. The lithium metal foil was used as the anode, and the 2025-type coin cell (Li/Li Cu(NO_3_)_2_) was assembled in the glove box.

### 2.2. Electrochemical Testing

For electrochemical testing, an Arbin BT2000 battery tester was used with a MITS Pro Arbin software. These tests were carried out at room temperature, and the cells were cycled between charge of 4.6 V and discharge of 2.3 V, at a C-rate of C/10 by applying a constant current of 350 μA. All the samples were tested for 10 charge and discharge cycles.

### 2.3. State of Charge (SOC) Measurements

The thermodynamics measurement system instrument ETMS and battery analyzer BA-1000 KVI PTE LTD, were applied to run the conditioning cycles. Cells were charged to 4.6 V under C/10 rate, and then a constant 4.6 V was applied until the current dropped below 0.1 mA. Following this, the cells were discharged to 2.3 V under C/10 rate, and a constant 2.3 V voltage was held until current dropped again below 0.1 mA. In this cycle, the ETMS determined the cells’ charge and discharge capacity.

### 2.4. Applied Equipment

**Figure 1:**
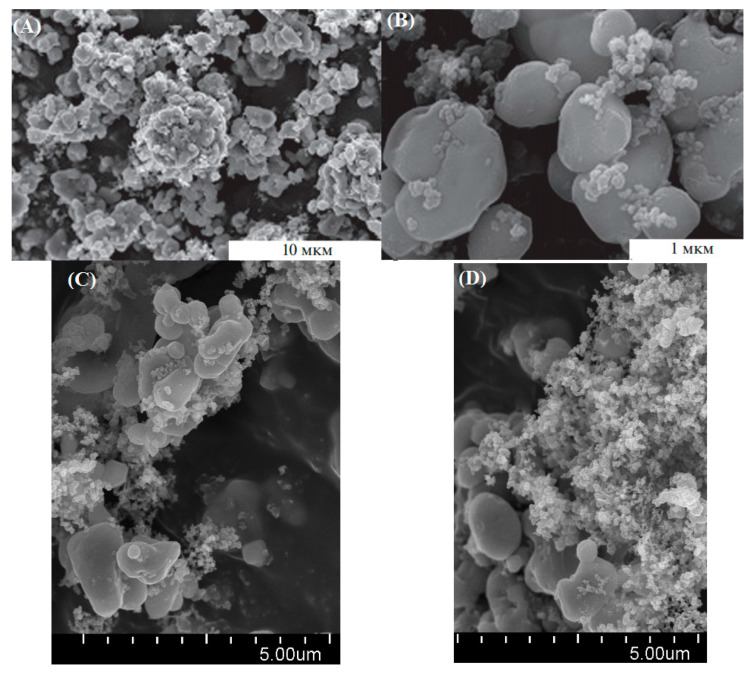
(**A**) Li_1.5_Cu_0.117_Co_0.366_Al_0.017_Mn_0.5_O_2_, (**B**) Li_1.667_Cu_0.1_Mg_0.017_Co_0.217_Mn_0.667_O_2_. (**C**) Li_1.667_Cu_0.117_Co_0.199_Al_0.017_Mn_0.667_O_2_, (**D**) Li_1.333_Cu_0.1_Mg_0.017_Co_0.551_Mn_0.333_O_2_.

The phase’s identities and crystal combination of all compositions were measured using single crystal X-ray diffraction ((SC-XRD)D8 QUEST ECO, Bruker AXS GmbH equipment from Germany, and their morphologies were analyzed with scanning electron microscopy (SEM) using a scanning electron microscope (Hitachi S-4000, Tokyo, Japan). In the cells, Li metal (99.9%, Aldrich Chem., Milwaukee, WI, USA) was used as anode and reference electrodes, 1 M LiPF6 in ethylene carbonate (EC)/diethylene carbonate (DEC) (1:1 mole ratio) as electrolyte, Celgard 2400 membrane (Hoechst Celanese Corp., Charlotte, NC, USA) as separator, and Li_2_MnO_3_-based composites as cathode. In the preparation of the composite cathodes, spinel powder was mixed with acetylene black (100%, Strem Chem., Newburyport, MA, USA). These tests were carried out to confirm the composition for evaluating the morphology of our synthesized composites (28 samples). These SEM morphologies are presented in suitable phases (between 1–5 microns for particle sizes) that are important for increasing the performance of the cathode materials (Figure 1).

### 2.5. X-ray Diffraction

**Figure 2:**
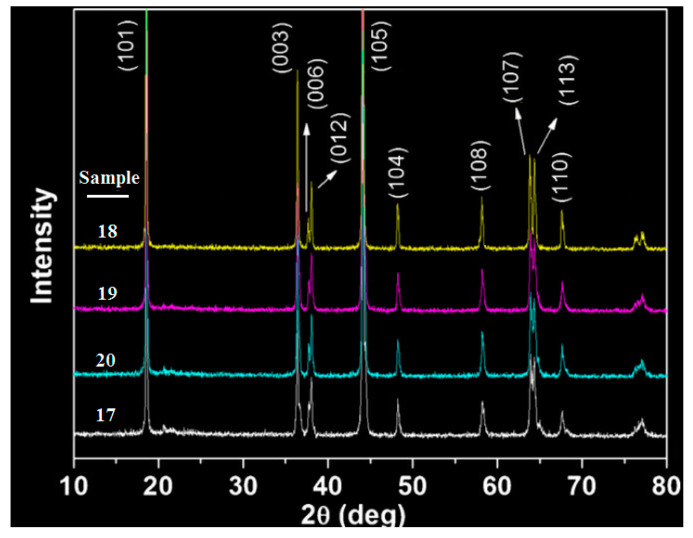
XRD patterns of the prepared cathode materials: Sample 17 = Li_1.667_Cu_0.117_Co_0.199_Al_0.017_Mn_0.667_O_2_; Sample 18 = Li_1.5_Cu_0.117_Co_0.366_Al_0.017_Mn_0.5_O_2_. Sample 19 = Li_1.333_Cu_0.117_Co_0.533_Al_0.017_Mn_0.333_O_2_; Sample 20 = Li_1.167_Cu_0.117_Co_0.699_Al_0.017_Mn_0.167_O_2_, cathode materials.

X-ray diffraction was performed for the 28 samples as well as for those which were repeated to distinguish whether the correct phase was achieved or not. X-ray photoelectron spectroscopy (Nexsa G2 XPS, Tianjin, China Model: TDS95-TDD250) and inductively coupled plasma atomic emission spectroscopy (Shimadzu ICPE, Kyoto, Japan) characterization techniques were only used on the samples that showed proper results and were repeated a number of times. XPS was carried out for a precise analysis on the percentage amount and the oxidations states of the elements present in the composition. ICP was performed for the detection of iron, titanium, manganese, and cobalt, as well as to verify the exact composition of the sample. X-ray diffraction was conducted on 28 items using a powder X-ray diffraction system that utilized Cu Kα radiation with λ = 1.55 Å. The θ angles in the X-rays, based on Bragg’s law, were compatible with the composites and sharp peaks that were observed. These sharp peaks were used for analyzing the structure of the 28 samples and lattice parameters, determining the formation of a pure phase, and for recognizing the existence of any unknown substances by comparing the observed diffraction data against a database maintained by the International Centre for diffraction data (Figure 2 and Table 4).

In addition, the inductively coupled plasma atomic emission spectroscopy (ICP-AES) technique was used for the identification of transition metals in the compositions of 28 samples. ICP analysis was carried out on 28 samples using a PerkinElmer Optima 7300 DV ICP-OES spectrophotometer from China with a Cetac ASX-520 auto sampler, Mein hard concentric nebulizer, and a cyclonic spray chamber with baffle. In addition, a statistical analysis for evaluating the most suitable cathode composites, in the viewpoint of capacity and cycle ability ranges, was completed for the 28 samples.

### 2.6. Charge and Discharge Measurement

The charge/discharge rates were measured using a BTS-610 battery tester from Korean with charge/discharge rates between 0.1 C and 0.4 C, in which C is equal to 170 mA g^−1^. During the testing, the cell was activated at 25 °C by 5 charge/discharge cycles. A charge of 4.5 V was followed by a discharge of 2.2 V; the cell was then soaked at 25° for 10 h to achieve thermal equilibrium. In the second step, the following charges and discharges were used: a charge of 4.5 V at 0.1 C, followed by a charge of 4.2 V for 3 h, and finally a discharge of 2.0 V. Afterwards, an electrochemical testing impedance spectroscopy measurement was made using an electrochemical analyzer model of CS310 Potentiostat with Electrochemical Impedance spectroscopy. Finally, the cells were disassembled, and a three-electrode system was assembled in a glove box. The LiFePO4 electrode was used as the working electrode, and lithium foils were used as the counter and reference electrodes. The signal of AC voltage of ±5 mV was used in frequency range between 102 and 105 Hz. The obtained impedance was fitted using ZSimpWin version 3.2 by EChem Software. 

## 3. Results and Discussion

This investigation was conducted to find the best cathode material compositions, including {[(1-x-y) LiCu_0.7_Co_0.3_] (Al and Mg doped)] O_2_}, xLi_2_MnO_3_, and yLiCoO_2_ systems, with high initial discharge capacity, great cyclability, and inexpensive cost compared to current lithium-ion cathode materials. Initially, 28 different composition points, according to the lever rule, stoichiometric weights, and mole-fractions, were chosen in order to find an optimized material with good electrochemical performance. These 28 points were extracted using the triangle phase diagram from the defined system (Scheme 1 and Table 2 and Table 3) and synthesis with the sol–gel method.

**Figure 3:**
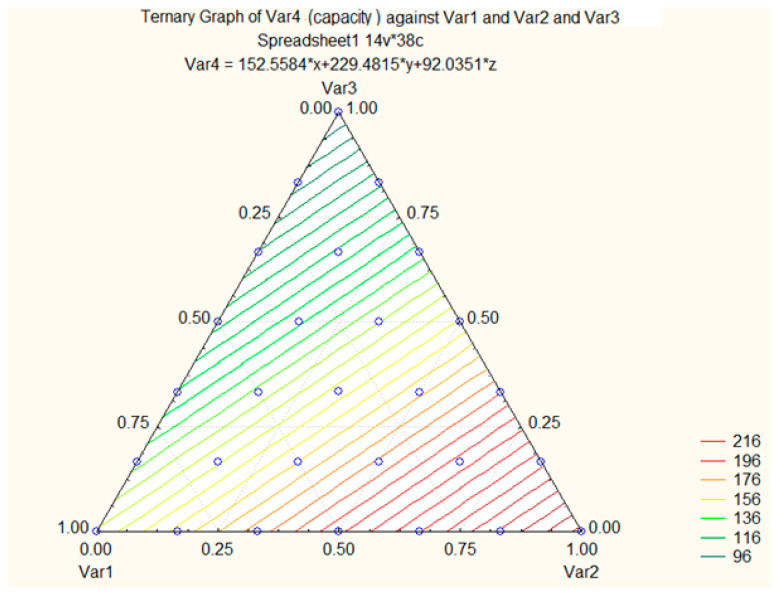
Ternary capacity versus Li_2_MnO_3_, Li_2_CoO_2_, and LiCu_0.7_Co_0.2_Al_0.1_O_2_ composites.

**Figure 4:**
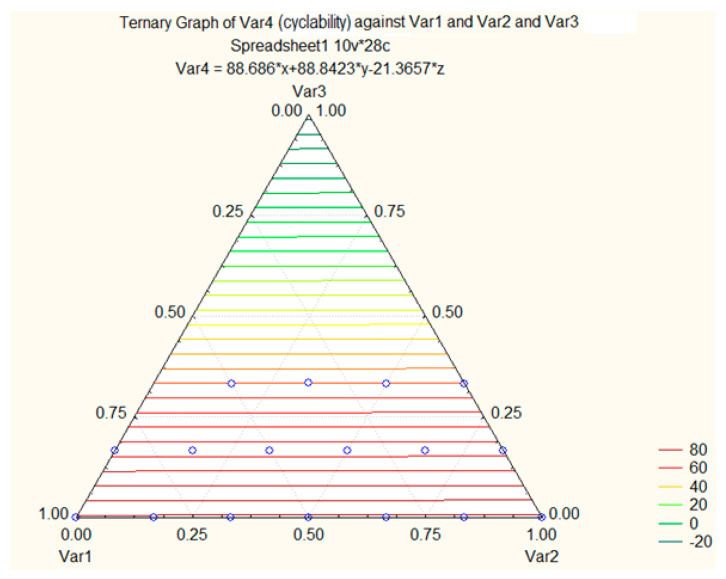
Ternary cyclability versus Li_2_MnO_3_, Li_2_CoO_2_, and LiCu_0.7_Co_0.2_Al_0.1_O_2_ composites.

Cu, Al and Mg amounts decreased toward the bottom of the triangle, while the percentages of Cu, Al and Mg were zero for Samples 22 and 24. A high Mn value was found in Sample 22, and its content decreased at the opposite end points of the triangle. Cobalt percentage was found in a wide region of the triangle and also decreased near Li (Li_0.33_Mn_0.66_) O_2_. It is predicted that the capacities and cyclability of the compositions are directly related to the amount of Mn, Co, Cu, Mg, and Al. The discharge capacity curves in this work were compared with the 18650-type “C/LiCoO_2_ Sony battery” result, which was modified by Ehrlich [21]. Since LiCoO_2_ cathode has an initial discharge capacity of around 145 mAh/g [22], any materials of these compositions with initial capacities of more than this amount (with a suitable cyclability of course) might be considered. The samples were tested with a cycler (Arbin BT 2000 battery testing system from China), between 2.5 V and 4.5 V with low C-rate of C/12. The initial results indicate a wide range and irregularity of cyclability and capacities. The initial discharge capacities varied from 102 mAh/g to 248 mAh/g. Both capacity and cyclability increased from LiCu_0.7_Co_0.3_Al_0.1_O_2_ toward the binary composition of Li_2_MnO_3_ and LiCoO_2_. Although Sample 20 showed a high capacity of 248.1 mAh/g, it contained low cyclability. Samples 25 and 18 exhibited good capacities of 201.2 and 220.2 mAh/g, respectively, with high cyclability, while due to the Mn^4+^ ion, Sample 22 had a low capacity. Although these types of data are not sufficient for determining a suitable cathode material in the viewpoint of capacity and cyclability amount, the statistical analysis can be useful for finding the region of best response from the data of the 28 samples. Therefore, testing with both capacity and cyclability relations, in consideration of the triangle regions, is needed. In this work, maintained by TIBCO software Inc., was used for analyzing the data in which Var_1_, Var_2_, and Var_3_ were Li_2_MnO_3_, Li_2_CoO_2_, and LiCu_0.7_Co_0.2_Al_0.1_O_2_, respectively, while Var_4_ was applied as the capacity in one test and as the cyclability in the other (Table 3 and Figure 3 and Figure 4).

Firstly, by using electrochemical analysis, among these compositions, three samples were chosen and subsequently synthesized, characterized, and tested. Using initial discharge capacity and some additional results in this study, we confirmed the high performance of two samples, namely, Sample 18, including Al doped with structure “Li_1.5_Cu_0.117_Co_0.366_Al_0.017_Mn_0.5_O_2_”, and Sample 17, including Mg doped with structure “Li_1.667_Ni_0.1_Mg_0.017_Co_0.217_Mn_0.667_O_2_”. When compared with other compositions, these samples were indicated as the best compositions among these 28 structures. Evidently, the used weight of cobalt in these samples was lower compared with that of LiCoO_2_, which is an advantage in the viewpoint of cost and reduced toxicity, which was the goal of this project.

**Figure 5:**
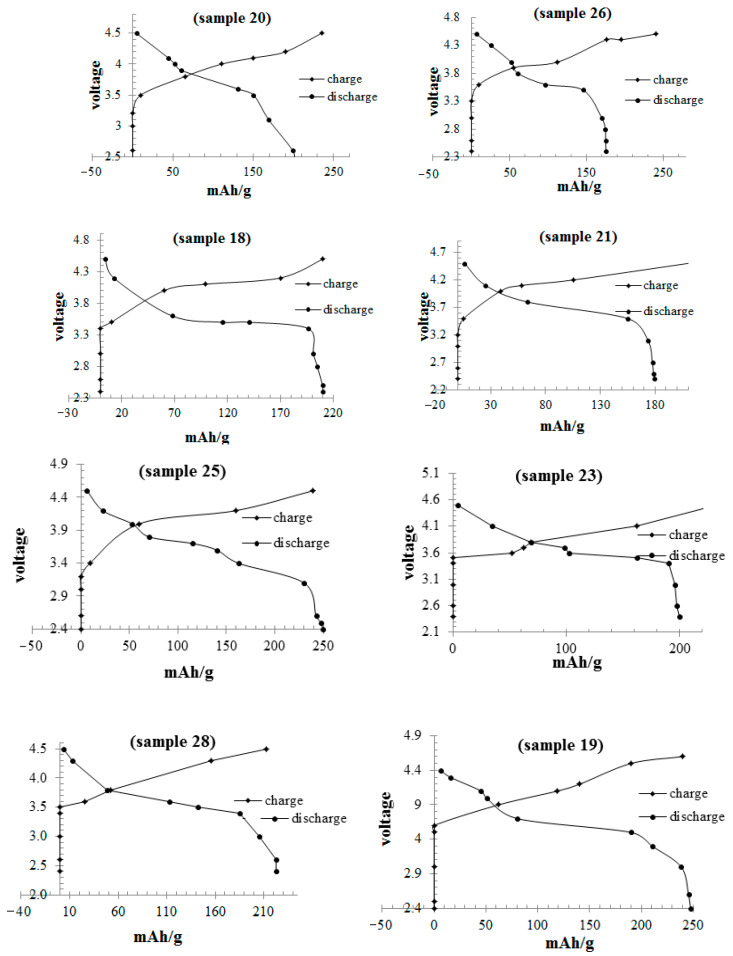
Charge and discharge diagrams of various samples.

Figure 5 presents the voltage-versus-capacity profiles of some of the samples for (1-x-y) LiCo_0.3_Cu_0.7_O_2_, xLi_2_MnO_3_, and yLiCoO_2_ compositions of cathode materials in the initial charge/discharge cycles. Although Sample 20 exhibited a high capacity (248.1 mAhg^−1^), it contained cyclability with a value of 52, which means it does not have a suitable composition for cathode material due to its low cyclability. Although Samples 27 and 28 (Table 3) exhibited suitable capacity and cyclability (235.5/98 and 250.2/99), respectively, they are not suitable for various reasons: firstly, they do not have ternary compositions, and, secondly, the percentages of cobalt in both samples are high, which is a disadvantage for the goal of this paper.

## 4. Conclusions

{[(1-x-y) LiCo_0.3_Cu_0.7_](Al and Mg doped)]O_2_}, xLi_2_MnO_3_, and yLiCoO_2_ cathodes with submicron particles were successfully synthesized through a sol–gel method. The structural and electrochemical properties were systemically investigated to examine the effects of charge/discharge capacities as well as capacity retention. The results show that the two prepared compounds, 1. Al doped with structure “Li_1.5_Cu_0.117_Co_0.366_Al_0.017_Mn_0.5_O_2_” and 2. Mg doped with structure Li_1.667_Ni_0.1_Mg_0.017_Co_0.217_Mn_0.667_O_2,_ when compared with other compositions with layer-type structures regardless of the copper content and due to the low percentages of Mg and Al, improved the capacity retention significantly. Moreover, this suppressed the phase transitions that usually occur in LiCuO_2_ during cycling and improved the charge/discharge reversibility of these two compositions. The percentage of copper and cobalt exhibited good performance. Although these types of system can help in eliminating the disadvantages of cobalt, which are mainly its cost and toxicity, the efficiency of these systems is similar to that of LiCoO_2_ cathode material. Therefore, the fabrication of lithium-ion batteries using more transition elements, such as Mn, Al, and Mg, is suggested for further research.

## Figures and Tables

**Table 1:** Characteristics of commercial Li-ion battery cathode materials [5].

Material	Potential vs. Li/Li^+^	Capacity (mAh/g)	Energy (Wh/Kg)	Advantage	Disadvantage
LiCoO_2_	3.95	140	546	Low	High
Cobalt, Nickel, Aluminum (CAN)	3.85	182–202	684–765	Slow reaction inside electrolyte High voltage and capacity Good performance	Expensive cost
Cobalt, Nickel, Manganese (CNM)	3.86	162–175	615–655	High capacity and operating voltages Slow reaction inside electrolyte Explosion	Expensive cost
LiMn_2_O_4_ (LMO)	4.14	105–125	415–495	Low safety due to oxygen release Suitable performance and voltage	Low cost
LiFePO_4_ (LFP)	3.40	155–175	515–585	Low voltage Low capacity	Low cost

**Table 2:** The 28 different compositions using the lever rule, stoichiometric weights, and mole-fractions of the triangle diagram.

Sample	Composition	Al Doped	Mg Doped
1	LiCu_0.7_Co_0.3_O_2_	LiCu_0.7_Co_0.2_Al_0.1_O_2_	LiCu_0.6_Mg_0.1_Co_0.3_O_2_
2	Li_1.167_Cu_0.583_Co_0.25_Mn_0.167_O_2_	Li_1.167_Cu_0.583_Co_0.167_Al_0.083_Mn_0.167_O_2_	Li_1.167_Cu_0.5_Mg_0.083_Co_0.25_Mn_0.167_O_2_
3	LiCu_0.583_Co_0.417_O_2_	LiCu_0.583_Co_0.334_Al_0.083_O_2_	LiCu_0.5_Mg_0.083_Co_0.42_O_2_
4	Li_1.333_Cu_0.467_Co_0.203_Mn_0.333_O_2_	Li_1.333_Cu_0.467_Co_0.133_Al_0.067_Mn_0.333_O_2_	Li_1.333_Cu_0.4_Mg_0.067_Co_0.2_Mn_0.333_O_2_
5	Li_1.167_Cu_0.467_Co_0.366_Mn_0.167_O_2_	Li_1.167_Cu_0.467_Co_0.299_Al_0.067_Mn_0.167_O_2_	Li_1.167_Cu_0.4_Mg_0.067_Co_0.366_Mn_0.167_O_2_
6	LiCu_0.467_Co_0.533_O_2_	LiCu_0.467_Co_0.466_Al_0.067_O_2_	LiCu_0.4_Mg_0.067_Co_0.533_O_2_
7	Li_1.5_Cu_0.35_Co_0.15_Mn_0.5_O_2_	Li_1.5_Cu_0.35_Co_0.1_Al_0.05_Mn_0.5_O_2_	Li_1.5_Cu_0.3_Mg_0.05_Co_0.15_Mn_0.5_O_2_
8	Li_1.333_Cu_0.35_Co_0.317_Mn_0.333_O_2_	Li_1.333_Cu_0.35_Co_0.267_Al_0.05_Mn_0.333_O_2_	Li_1.333_Cu_0.3_Mg_0.05_Co_0.317_Mn_0.333_O_2_
9	Li_1.167_Cu_0.35_Co_0.483_Mn_0.167_O_2_	Li_1.167_Cu_0.35_Co_0.433_Al_0.05_Mn_0.167_O_2_	Li_1.167_Cu_0.3_Mg_0.05_Co_0.483_Mn_0.167_O_2_
10	Li Cu_0.35_Co_0.6_O_2_	LiCu_0.35_Co_0.6_Al_0.05_O_2_	LiCu_0.3_Mg_0.05_Co_0.65_O_2_
11	Li_1.667_Cu_0.233_Co_0.1_Mn_0.667_O_2_	Li_1.667_Cu_0.233_Co_0.067_Al_0.033_Mn_0.667_O_2_	Li_1.667_Cu_0.2_Mg_0.033_Co_0.1_Mn_0.667_O_2_
12	Li_1.5_Cu_0.233_Co_0.267_Mn_0.5_O_2_	Li_1.5_Cu_0.233_Co_0.234_Al_0.033_Mn_0.5_O_2_	Li_1.5_Cu_0.2_Mg_0.033_Co_0.267_Mn_0.5_O_2_
13	Li_1.333_Cu_0.233_Co_0.434_Mn_0.333_O_2_	Li_1.333_Cu_0.233_Co_0.4_Al_0.033_Mn_0.333_O_2_	Li_1.333_Cu_0.2_Mg_0.033_Co_0.434_Mn_0.333_O_2_
14	Li_1.167_Cu_0.233_Co_0.6_Mn_0.167_O_2_	Li_0.167_Cu_0.233_Co_0.567_Al_0.033_Mn_0.167_O_2_	Li_1.167_Cu_0.2_Mg_0.033_Co_0.6_Mn_0.167_O_2_
15	LiCu_0.233_Co_0.767_O_2_	LiCu_0.233_Co_0.734_Al_0.033_O_2_	LiCu_0.2_Mg_0.033_Co_0.767_O_2_
16	Li_1.835_Cu_0.116_Co_0.049_Mn_0.835_O_2_	Li_1.833_Cu_0.117_Co_0.033_Al_0.017_Mn_0.833_O_2_	Li_1.835_Cu_0.1_Mg_0.017_Co_0.049_Mn_0.835_O_2_
17	Li_1.667_Cu_0.116_Co_0.217_Mn_0.667_O_2_	Li_1.667_Cu_0.117_Co_0.199_Al_0.017_Mn_0.667_O_2_	Li_1.667_Cu_0.1_Mg_0.017_Co_0.217_Mn_0.667_O_2_
18	Li_1.5_ Cu_0.116_Co_0.384_Mn_0.5_O_2_	Li_1.5_Cu_0.117_Co_0.366_Al_0.017_Mn_0.5_O_2_	Li_1.5_Cu_0.1_Mg_0.017_Co_0.384_Mn_0.5_O_2_
19	Li_1.333_Cu_0.116_Co_0.551_Mn_0.333_O_2_	Li_1.333_Cu_0.117_Co_0.533_Al_0.017_Mn_0.333_O_2_	Li_1.333_Cu_0.1_Mg_0.017_Co_0.551_Mn_0.333_O_2_
20	Li_1.167_Cu_0.116_Co_0.717_Mn_0.167_O_2_	Li_1.167_Cu_0.117_Co_0.699_Al_0.017_Mn_0.167_O_2_	Li_1.167_Cu_0.1_Mg_0.017_Co_0.717_Mn_0.167_O_2_
21	LiCu_0.116_Co_0.884_O_2_	LiCu_0.117_Co_0.866_Al_0.017_O_2_	LiCu_0.1_Mg_0.017_Co_0.884_O_2_
22	Li_2_MnO_2_	Li_2_MnO_2_	Li_2_MnO_2_
23	Li_1.833_Co_0.167_Mn_0.833_O_2_	Li_1.833_Co_0.167_Mn_0.833_O_2_	Li_1.833_Co_0.167_Mn_0.833_O_2_
24	Li_1.667_Co_0.333_Mn_0.667_O_2_	Li_1.667_Co_0.333_Mn_0.667_O_2_	Li_1.667_Co_0.333_Mn_0.667_O_2_
25	Li_1.5_Co_0.5_Mn_0.5_O_2_	Li_1.5_Co_0.5_Mn_0.5_O_2_	Li_1.5_Co_0.5_Mn_0.5_O_2_
26	Li_1.333_ Co_0.667_Mn_0.333_O_2_	Li_1.333_Co_0.667_Mn_0.333_O_2_	Li_1.333_Co_0.667_Mn_0.333_O_2_
27	Li_1.167_Co_0.833_Mn_0.167_O_2_	Li_1.167_Co_0.833_Mn_0.167_O_2_	Li_1.167_Co_0.833_Mn_0.167_O_2_
28	LiCoO_2_	LiCoO_2_	LiCoO_2_

**Table 3:** Summary of capacity and cyclability for 28 samples of {[(1-x-y) LiCo_0.3_Cu_0.7_](Al doped)]O_2_}, xLi_2_MnO_3_, and yLiCoO_2_ composites.

Samples	Li_2_MnO_3_	LiCoO_2_	LiCu_0.7_Co_0.3_(Al Doped)]O_2_	Blend	Capacity	Cyclability
1-	0	0	1	Pure	102.5	81
2-	1/6 = 0.167	0	0.833	Binary	95.5	67
3-	0	0.167	0.833	Binary	98.5	73
4-	1/3 = 0.333	0	0.667	Binary	135.5	90
5-	1/6 = 0.167	0.167	0.667	Ternary	130.5	82
6-	0	0.333	0.667	Binary	96.5	71
7-	1/2 = 0.500	0	0.500	Binary	152.5	75
8-	1/3 = 0.333	0.167	0.500	Ternary	179.5	68
9-	1/6 = 0.167	0.333	0.500	Ternary	156.5	83
10-	0	0.500	0.500	Binary	171.5	99
11-	2/3 = 0.667	0	0.333	Binary	102.6	95
12-	1/2 = 0.500	0.167	0.333	Ternary	153.1	90
13-	1/3 = 0.333	0.333	0.333	Ternary	173.4	78
14-	1/6 = 0.167	0.500	0.333	Ternary	174.5	83
15-	0	0.667	0.333	Binary	116.8	93
16-	5/6 = 0.833	0	0.167	Binary	182.5	81
17-	2/3 = 0.667	0.167	0.167	Ternary	173.6	95
18-	1/2 = 0.500	0.333	0.167	Ternary	250.5	105
19-	1/3 = 0.333	0.5	0.167	Ternary	137.5	93
20-	1/6 = 0.167	0.667	0.167	Ternary	248.1	52
21-	0	0.833	0.167	Binary	181.5	93
22-	1	0	0	Pure	71.6	96
23-	5/6 = 0.833	0.167	0	Binary	181.5	91
24-	2/3 = 0.667	0.333	0	Binary	174.2	95
25-	1/2 = 0.500	0.500	0	Binary	193.5	92
26-	1/3 = 0.333	0.667	0	Binary	192.5	58
27-	1/6 = 0.167	0.833	0	Binary	235.5	98
28-	0	1	0	Pure	250.2	99

**Table 4:** Lattice parameters of Samples 14, 16, 18, and 21.

Sample	a (nm)	b (nm)	C (nm)	Capacity	Cyclability
17 (Ternary)	0.2215	0.2432	1.012	171.5	85
18 (Ternary)	0.2435	0.2546	1.093	250.5	105
19 (Ternary)	0.2923	0.2612	1.048	220.2	95
20 (Ternary)	0.3041	0.2883	1.422	185.5	96

## References

[1] (2010). Lithium Ion Batteries Outlook and Alternative Energy Vehicle (HEVs, PHEVs) Technologies, Markets, Competitors and Opportunities: 2010–2012.

[2] Yoshio M., Brodd R.J., Kozawa A. (2009). Lithium-Ion Batteries: Science and Technologies.

[3] Scrosati B., Garche J. (2010). Lithium Batteries: Status, Prospects and Future. J. Power Sources.

[4] Daniel C., Besenhard J.O. (2011). Handbook of Battery Materials.

[5] Meyers R.A. (2012). Encyclopedia of Sustainability Science and Technology.

[6] Rossen E., Jones C., Dahn J. (1992). Structure and Electrochemistry of Li_x_MnyNi_1−y_O_2_. Solid State Ion..

[7] Spahr M.E. (1998). Characterization of Layered Lithium Nickel Manganese Oxides Synthesized by a Novel Oxidative Coprecipitation Method and Their Electrochemical Performance as Lithium Insertion Electrode Materials. J. Electrochem. Soc..

[8] Ohzuku T., Makimura Y. (2001). Layered Lithium Insertion Material of LiNi_1/2_Mn_1/2_O_2_: A Possible Alternative to LiCoO_2_ for Advanced Lithium-Ion Batteries. Chem. Lett..

[9] Makimura Y., Ohzuku T. (2003). Lithium insertion material of LiNi_1/2_Mn_1/2_O_2_ for advanced lithiumion batteries. J. Power Sources.

[10] Liu Z., Yu A., Lee J. (1999). Synthesis and characterization of LiNi_1−x−y_CoxMnyO_2_ as the cathode materials of secondary lithium batteries. J. Power Sources.

[11] Yoshio M., Noguchi H., Itoh J.I., Okada M., Mouri T. (2000). Preparation and properties of LiCoyMnxNi_1−x−y_O_2_ as a cathode for lithium ion batteries. J. Power Sources.

[12] Ohzuku T., Makimura Y. (2001). Layered Lithium Insertion Material of LiCo_1/3_Ni_1/3_Mn_1/3_O_2_ for Lithium-Ion Batteries. Chem. Lett..

[13] Belharouak I., Sun Y.K., Liu J., Amine K. (2003). Li(Ni_1/3_Co_1/3_Mn_1/3_)O_2_ as a suitable cathode for high power applications. J. Power Sources.

[14] Ngala J.K., Chernova N.A., Ma M., Mamak M., Zavalij P.Y., Whittingham M.S. (2004). The synthesis, characterization and electrochemical behavior of the layered LiNi_0.4_Mn_0.4_Co_0.2_O_2_ compound. J. Mater. Chem..

[15] Yoon W.S., Chung K.Y., McBreen J., Yang X.Q. (2006). A comparative study on structural changes of LiCo_1/3_Ni_1/3_Mn_1/3_O_2_ and LiNi_0.8_Co_0.15_Al_0.05_O_2_ during first charge using in situ XRD. Electrochem. Commun..

[16] Ammundsen B., Paulsen J., Davidson I., Liu R.S., Shen C.H., Chen J.M., Jang L.Y., Lee J.F. (2002). Redox Mechanism of Layered Li_1.2_Cr_0.4_Mn_0.4_O_2_ Cathode Material. J. Electrochem. Soc..

[17] Thackeray M.M., Johnson C.S., Vaughey J.T., Li N., Hackney S.A. (2005). Advances in manganese-oxide ‘composite’ electrodes for lithium-ion batteries. J. Mater. Chem..

[18] Zhang L., Noguchi H., Yoshio M. (2002). Synthesis and electrochemical properties of layered Li–Ni–Mn–O compounds. J. Power Sources.

[19] Shin S.S., Sun Y.K., Amine K. (2002). Synthesis and electrochemical properties of Li[Li_(1−2x)/3_Ni_x_Mn_(2−x)/3_]O_2_ as cathode materials for lithium secondary batteries. J. Power Sources.

[20] Zhang H. (2014). Synthesis and Characterization of Li Ni_0.7-x_ Mg_x_Co_0.3_O_2_ (0 ≤ x ≤ 0.1) Cathode Materials for Lithium-Ion Batteries Prepared by a Sol-Gel Method. Adv. Mater. Sci. Eng..

[21] Ehrlich G.M., Linden D. (2002). Handbook of Lithium-Ion Batteries.

[22] Yang Z.-G., Zhang J.-L., Kintner-Meyer M.C.W., Lu X.-C., Choi D.-W., Lemmon J.P., Liu J. (2011). Electrochemical Energy Storage for Green Grid. Chem. Rev..

